# Human papillomavirus self‐testing among unscreened and under‐screened Māori, Pasifika and Asian women in Aotearoa New Zealand: A preference survey among responders and interviews with clinical‐trial nonresponders

**DOI:** 10.1111/hex.13599

**Published:** 2022-09-26

**Authors:** Susan M. Sherman, Naomi Brewer, Karen Bartholomew, Collette Bromhead, Sue Crengle, Chris Cunningham, Jeroen Douwes, Sunia Foliaki, Jane Grant, Anna Maxwell, Georgina McPherson, Nina Scott, Helen Wihongi, John D. Potter

**Affiliations:** ^1^ School of Psychology Keele University Keele UK; ^2^ Research Centre for Hauora and Health Massey University Wellington Aotearoa New Zealand; ^3^ Waitematā District Health Board (DHB) Auckland New Zealand; ^4^ Auckland DHB Auckland New Zealand; ^5^ School of Health Sciences Massey University Wellington Aotearoa New Zealand; ^6^ Department of Preventive and Social Medicine University of Otago Dunedin Aotearoa New Zealand; ^7^ Hei Āhuru Mōwai

**Keywords:** acceptability, Asian women, HPV, Māori women, Pasifika women, self‐sample, self‐test

## Abstract

**Introduction:**

Māori, Pasifika and Asian women are less likely to attend cervical screening and Māori and Pasifika women are more likely to be diagnosed with later‐stage cervical cancer than other women in Aotearoa New Zealand. This study—with under‐screened women taking part in a randomized‐controlled trial comparing self‐testing and standard screening—explored the acceptability of a human papillomavirus (HPV) self‐test kit and the preferred method for receiving it.

**Methods:**

Māori, Pasifika and Asian women (*N*= 376) completed a cross‐sectional postal questionnaire. Twenty‐six women who had not accepted the trial invitation were interviewed to understand their reasons for nonparticipation.

**Results:**

Most women found the self‐test kit easy and convenient to use and reported that they did not find it painful, uncomfortable or embarrassing. This was reflected in the preference for a self‐test over a future smear test on the same grounds. Most women preferred to receive the kit by mail and take the test themselves, rather than having it done by a doctor or nurse. There was a range of preferences relating to how to return the kit. Phone calls with nonresponders revealed that, although most had received the test kit, the reasons for not choosing to be involved included not wanting to, being too busy or forgetting.

**Conclusion:**

HPV self‐testing was acceptable for Māori, Pasifika and Asian women in Aotearoa New Zealand. HPV self‐testing has considerable potential to reduce the inequities in the current screening programme and should be made available with appropriate delivery options as soon as possible.

**Patient or Public Contribution:**

This study explored the acceptability of HPV self‐testing and their preferences for engaging with it among Māori, Pasifika and Asian women. Thus, women from these underserved communities were the participants and focus of this study.

## 1 INTRODUCTION

Globally, cervical cancer is the fourth most common cancer affecting women, with around 570,000 diagnoses and 311,000 deaths in 2018.^1^ In Aotearoa New Zealand, there were 190 diagnoses and 72 deaths in 2018; cervical cancer is the fourth most common cancer in women aged 15–44 years.^2^ Māori women have a 1.7× higher incidence of this cancer (age‐standardized rate of 9.4 cases per 100,000) than non‐Māori women (5.4 cases per 100,000), and the mortality rate is 2.7 times higher for Māori women (3.0 deaths per 100,000) than non‐Māori women (1.1 cases per 100,000).^3^ A review of cervical cancer incidence in 2013–2017^4^ showed that, for those women who were screened in the 6–84 months before diagnosis, 40% of Māori women and 53% of Pasifika* women had a high‐grade cervical cytology sample compared to 16% of European women, suggesting missed opportunities for prevention or earlier diagnosis. One of the key recommendations of the review was that resources be ‘provided to *improve access to screening* and treatment of cervical precancer for Māori women…. *Intervention strategies should take into consideration both the practical and cultural needs* of these women/wāhine’^4^, ^p.14^ (emphasis added).

High‐risk human papillomavirus (HPV) types, primarily transmitted by sexual contact, are detected in more than 90% of cervical cancers.^6^ Persistent infection with high‐risk HPV can result in precancerous changes in cervical cells, which, if left untreated, can progress to cervical cancer. Currently, the National Cervical Screening Programme (NCSP) in Aotearoa New Zealand recommends cervical screening every 3 years for women aged 25–69 years, with cytology as the primary test.^7^ HPV testing is an alternative primary screening test and many countries have already implemented this in their programmes.

The overall 3‐year screening coverage in Aotearoa New Zealand decreased from 72.2% in March 2019 to 70.2% in March 2021^8^ against a target of 80%. For Māori women, the decrease was from 64.6% to 61.2%; for Pasifika women, it was 69.4%–63.1%; and for Asian women, it was 62.4%–61.4%. The existing inequitable outcomes for Māori and Pasifika women are likely to worsen if these declines in screening continue. Moving to a system of primary HPV screening provides the possibility of offering women the option of HPV self‐testing. This can be done in women's homes or in a clinic setting and may address patient/client‐ and provider‐related barriers to screening that have arisen due to structural and systemic biases.

A previous study that explored barriers to cervical screening for Māori women, as well as hypothetical acceptability of HPV self‐testing,^9^ found that the primary barriers to conventional, more invasive screening were lack of time/other commitments, fear of discomfort or pain and that some Māori women consider this area to be tapu (sacred) and, therefore, in some cases, screening caused feelings of embarrassment.† In that study, 61.2% of women said that they would prefer an HPV self‐test to either a clinician‐taken HPV test or conventional screening and 73.3% said they would be likely or very likely to self‐test if it were offered. A pilot study exploring acceptability found that 66% of under‐screened Māori and Pasifika women preferred to self‐test after trying a device.^10^ Subsequently, two randomized‐controlled trials (RCTs) have been conducted to explore HPV self‐testing with under‐screened or never‐screened women, both with a focus on Māori. In the first study, women in the intervention arm of the RCT were offered an HPV self‐test, although they could opt instead for a clinician‐taken sample or a smear test. The self‐test could be done at home, in the clinic or at a community centre. The control arm was usual care, so the women were offered a clinician‐taken smear test. Of women in the self‐test arm of the RCT, 59% were screened, compared to 22% of those in the usual‐care arm, suggesting that an offer of self‐testing could substantially increase screening uptake among Māori women.^11^

A second, larger, RCT conducted by our research team^10^ was preceded by a feasibility study,^12^ which focused on stakeholder codesign and testing the cultural appropriateness of materials. Between 2018 and 2020, an open‐label, three‐arm, community‐based, randomized‐controlled trial in which unscreened or under‐screened (≥5 years overdue) Māori, Pasifika and Asian women from Auckland were invited for cervical screening was undertaken. The three trial arms were as follows: *usual care*, in which women were invited to attend a clinic for a standard smear sample; *clinic‐based self‐testing*, in which women were invited to take a self‐test at their usual general practice; and *mail‐out self‐testing*, in which women were posted a kit and invited to take a self‐test at home. This showed that, although screening uptake was lower than in the first RCT, self‐testing uptake was again statistically significantly preferred over usual care, with the highest participation in the mail‐out self‐test arm.^13^

Here, we report the findings from the acceptability survey that was given to women participating in the self‐testing arms of the second RCT and nested sub‐study in which some nonresponding women were offered opportunistic self‐sampling when they presented to the clinic for other reasons,^10^ as well as the findings from a telephone survey conducted with nonresponding women. The main aims of the survey and interviews were to explore the acceptability of the HPV self‐test‡ kit; to determine what preferences women had for invitation, sample return and follow‐up methods; to establish whether the level of information in the participant material was appropriate and acceptable for Māori, Pasifika and Asian women; and to determine whether further localization or refinement was required.

## 2 METHOD

### 2.1 Design

We conducted a paper‐based cross‐sectional survey throughout the main trial and nested sub‐study, which recruited women between June 2018 and May 2020. We also conducted telephone interviews with women who did not respond to the invitation to take part in the trial, to understand their reasons for nonparticipation.

### 2.2 Participants

All Māori, Pasifika and Asian women who were invited to take part in one of the two self‐testing arms of the RCT^13^ were given a survey to complete about their experience of self‐testing, which they completed at home or in the clinic, depending on where they completed the self‐testing. The women were all aged 30‐69 years, were resident in Waitematā or Auckland District Health Board (DHB) areas and had never been screened or were overdue (≥5 years) for cervical screening.

Before beginning this trial, we completed a feasibility and acceptability study, where we conducted ethnic‐specific focus groups with a health‐literacy expert to develop and improve the information and its presentation.^12^ Women were identified through a routinely available national data‐match process between Primary Health Organizations (organizations responsible for primary care) and the NCSP, where the screening status of enroled women is updated monthly.^10^ They were deemed to be unscreened or under‐screened if no screening had been recorded for the last 5 years.

Women were invited through their usual primary care provider (in partnership with the research team). Women in the self‐testing at home arm were posted the kit, instructions and reply envelope along with the questionnaire. The information sent included a link to the webpage with translated study documents and the study video clips. Women in the clinic‐based self‐testing arm were posted an invitation to the clinic with booking details, completed nurse consent when they attended the clinic and completed the paper‐based questionnaire at the time of the test. Women in the opportunistic sub‐study were sent a letter or text informing them that they were now able to self‐test at their clinic or request that a self‐testing kit be mailed to them, both for a limited period of time. In addition, an alert was put on the primary care dashboard so that if they attended for any other reason, they would be offered a self‐test in the study period.

A random sample of women who were invited to the RCT but did not take part (nonresponders) was contacted by telephone to take part in a telephone interview. The aim was to recruit equal numbers of women from each ethnicity and study group.

### 2.3 Measures—Survey

The survey items were localized and developed (based on feedback) from the Australian iPap study responders' posttest questionnaire^14^ with permission from the iPAP investigators and these, along with some additional questions, are detailed below.

#### 2.3.1 Questions about the cervical screening test

All women were asked about their experience of the self‐test kit; whether the instructions were clear and easy to understand; whether they had watched the study video clips (the women were directed to a webpage with clips that had subtitles in several languages: Te Reo Māori, Tongan, Sāmoan, Korean and Simplified Chinese); if so, which ones; and for any other comments.

Women who had previously undergone a smear test were asked questions comparing their experience of the self‐test kit with their experience of a smear test.

All women were presented with a list of 19 possible reasons^10^ why they might not have had a smear test recently—or ever; they were asked to identify all the reasons that applied, to further identify their main reason and to provide any additional reason(s).

Women were asked questions regarding how they would like to have a cervical screening test in the future. They were asked to identify their two main reasons for this choice from a list of six options, which varied depending on whether they would prefer to have a test taken by a doctor/nurse or to receive a self‐test kit. If relevant, they were asked follow‐up questions exploring preferences for the collection and return of the self‐test kit. Finally, they were asked whether they would recommend the self‐test kit to a friend or whānau/family member and whether they would be more likely to take part in regular screening if they were able to do the test themselves.

#### 2.3.2 Socio‐demographic questions

The final part of the survey included a series of socio‐demographic questions regarding highest level of schooling; household income; which generation of their family came to Aotearoa New Zealand; whether English is their first language; and whether they identify as Māori.

### 2.4 Measures—Interviews

A structured interview guide was used with nonresponders to explore whether they had received the invitation to the trial and their reasons for not taking part as detailed in Table 1.

**Table 1:** Interview guide for nonresponders to the clinical trial

Question	Example script	Action
Did they receive the invitation?	*I'm calling from the Women's Health self‐test study—did you receive the invitation we sent you?*	If no, brief explanation and offer to send kit.
		Update address details
If yes, enquire into their response to the invitation for example:	*Did you get a chance to have a look at it?*	Record any comments.
Was the invitation clear?	*How did you find the info we sent you?*	Offer follow‐up call from nurse if appropriate.
Did they understand what they were being asked to do?	*Was it clear what you were being invited to do?*	
Did they decide not to participate?	*Was there a reason that you didn't do the self‐test?*	
If appropriate, find out what would have made them participate.	*Do you have any suggestions?*	Record any comments.
	*Is there anything we could have done differently?*	

### 2.5 Analysis

The survey data are summarized using descriptive statistics. We ran a series of Pearson's *χ* ^2^ tests to compare the reasons for not ever or not recently having had a smear test across ethnicities.

To analyse the open‐ended responses to survey questions and the telephone interviews, we undertook content analysis using an emergent‐coding approach, whereby codes were identified from the data rather than a priori.^15^ The interview data consist of short accounts of the conversation documented by the Māori interviewer making the call.

Ethnicity data were collected and categorized in line with the HISO 10001:2017 Ethnicity Data Protocols, whereby respondents self‐identify and responses are categorized according to Statistics NZ descriptors (https://www.health.govt.nz/publication/hiso-100012017-ethnicity-data-protocols). We used a prioritized output (if multiple ethnicities are identified, only one is included in the analyses), ordered Māori >  Pasifika > Asian > Other, as per the Ethnicity‐data standards.

## 3 RESULTS

The results reported below are from those women who responded to the invitation to take part in the study.

### 3.1 Interviews with nonresponders

Interviews with nonresponders were also conducted. One hundred and twelve contact attempts were made, at different times of the day. In total, 26 women were interviewed; 16 of these were from the self‐test in the clinic group (Māori, *N*= 4; Pasifika, *N* = 4; Asian, *N* = 6) and 12 were from the self‐test at home group (Māori, *N* = 4; Pasifika, *N* = 4; Asian, *N* = 4). The content analysis of the notes made by the interviewer generated 25 unique codes. The codes along with a frequency count are presented in Supporting Information: Table S3.

### 3.2 Survey

In total, 376 women (75.7% of women who returned their test) completed the survey (mean age, 46.5 years; SD = 10.6; range, 30–71). Participants' characteristics are presented in Table 2.

**Table 2:** Characteristics of women who completed the survey

Demographic questions	Level	*n* (%)
Ethnicity	Māori	108 (28.7)
	Pasifika	105 (27.9)
	Asian	163 (43.4)
Which generation of family came to New Zealand?	Born in Aotearoa New Zealand	132 (35.1)
	Moved to New Zealand from another country	174 (46.3)
	Parents moved to New Zealand	24 (6.4)
	Grandparents moved to New Zealand	8 (2.1)
	Family moved to New Zealand before grandparents were born	4 (1.1)
	Prefer not to say	8 (2.1)
	Left blank	26 (6.9)
English is the first language	Yes	183 (48.7)
	No	162 (43.1)
	Left blank	31 (8.2)
Highest level of schooling	Primary school	9 (2.4)
	Secondary school (college)	105 (27.9)
	Technical or trade school diploma	56 (14.9)
	Undergraduate university degree	89 (23.7)
	Postgraduate university degree	83 (22.1)
	None	8 (2.1)
	Left blank	26 (6.9)
Household's approximate gross (before tax, levies, etc.) annual income	$1–$20,000	27 (7.2)
	$20,001–$50,000	81 (21.5)
	$50,001–$70,000	48 (12.8)
	$70,001–$100,000	59 (15.7)
	$100,001–$150,000	32 (8.5)
	$150,001 or more	26 (6.9)
	Prefer not to say	66 (17.6)
	Left blank	37 (9.8)

### 3.3 Using the self‐test kit

Women were asked to indicate the extent to which they agreed with a series of statements about the HPV self‐test kit. The responses are presented in Table 3.

**Table 3:** Women's responses to statements about using the self‐test kit (%)

	Not at all	A little	Very much	Unsure/do not know	Total *N*
It was easy to use the swab					
Māori	6 (5.7)	10 (9.5)	88 (83.8)	1 (1.0)	105
Pasifika	4 (3.9)	5 (4.9)	91 (89.2)	2 (2.0)	102
Asian	3 (1.9)	23 (14.5)	132 (83.0)	1 (0.6)	159
Total	13 (3.6)	38 (10.4)	311 (85.0)	4 (1.1)	366
Taking the test using the swab was painful***					
Māori	96 (90.6)	6 (5.7)	3 (2.8)	1 (0.9)	106
Pasifika	78 (85.7)	12 (13.2)	1 (1.1)	0 (0.0)	91
Asian	119 (75.8)	37 (23.6)	1 (0.6)	0 (0.0)	157
Total	293 (82.8)	55 (15.5)	5 (1.4)	1 (0.3)	354
Taking the test using the swab was uncomfortable***					
Māori	84 (80.8)	19 (18.3)	1 (1.0)	0 (0.0)	104
Pasifika	70 (76.1)	20 (21.7)	2 (2.2)	0 (0.0)	92
Asian	97 (62.6)	56 (36.1)	2 (1.3)	0 (0.0)	155
Total	251 (71.5)	95 (27.1)	5 (1.42)	0 (0.0)	351
I felt embarrassed					
Māori	98 (93.3)	6 (5.7)	1 (1.0)	0 (0.0)	105
Pasifika	82 (89.1)	8 (8.7)	1 (1.1)	1 (1.1)	92
Asian	135 (86.5)	18 (11.5)	3 (1.9)	0 (0.0)	156
Total	315 (89.2)	32 (9.1)	5 (1.4)	1 (0.3)	353
It was convenient***,^a^					
Māori	20 (18.7)	2 (1.9)	85 (79.4)	0 (0.0)	107
Pasifika	16 (17.4)	4 (4.3)	69 (75.0)	3 (3.3)	92
Asian	25 (15.8)	17 (10.8)	114 (72.2)	2 (1.3)	158
Total	61 (17.1)	23 (6.4)	268 (75.1)	5 (1.4)	357
I am confident I did it correctly					
Māori	10 (9.6)	15 (14.4)	75 (72.1)	4 (3.8)	104
Pasifika	9 (9.6)	10 (10.6)	69 (73.4)	6 (6.4)	94
Asian	12 (7.6)	29 (18.5)	100 (63.7)	16 (10.2)	157
Total	31 (8.7)	54 (15.2)	24 (68.7)	26 (7.3)	355

a Feedback from Research Nurses who were with women completing the questionnaire suggested that the high number of women reporting ‘not at all convenient’ was due to a misunderstanding about the question.

^***^ *p* < .05 indicating a statistically significant effect of ethnicity on responses using a 3(ethnicity) × 4(response type) Pearson *χ* ^2^ test.

In an additional question, of the 354 women who answered the question, 99.4% (*N* = 352) said that the self‐test kit instructions were clear and easy to understand, with only 2 women indicating that they were not. Of the 358 women who answered the question, 78.5% (*N* = 281) said that they had not watched the video clips, while 21.5% (*N* = 77) said they had watched the video clips. The videos watched were About the HPV self‐test study (*N*= 59); How to take part in the study (*N* = 50); How to do the test (*N* = 58); Getting your test results (*N* = 44); and About Cervical Screening And Your Rights (*N* = 44). Sixty‐seven women indicated that the video clips were helpful, with just five women saying they were not.

Forty‐two women provided responses other than N/A or ‘I didn't watch them’ to the open‐ended question asking for comments about the video clips (see the Supporting Information).

### 3.4 Comparison of the self‐test kit with the previous smear test

Women who had previously had a smear test were asked to compare this with the self‐test kit and their responses are presented in Supporting Information: Table S1. The self‐test was found to be easier, more convenient, less embarrassing and less uncomfortable by all groups. Women were less clear about the comparative accuracy of the two tests.

### 3.5 Barriers to smear test

The number and percentage of reasons for not ever or not recently having had a smear test are presented in Table 4.

**Table 4:** Reasons for not ever or not recently having had a smear test (%)

Question	Māori	Pasifika	Asian	*N* (%^a^)
A test from a nurse or doctor is embarrassing^b^	60 (55.6)	47 (44.8)	57 (35.0)	164 (43.6)***
A smear test from a nurse or doctor is too painful or uncomfortable^b^	40 (37.0)	35 (33.3)	47 (28.8)	122 (32.4)***
It is hard to find the time to have a test^b^	33 (30.6)	37 (35.2)	49 (30.1)	119 (31.6)***
I don't think I need a test	17 (15.7)	19 (18.1)	41 (25.2)	77 (20.5)
I don't feel comfortable asking for a test from my nurse or doctor	29 (26.9)	21 (20.0)	24 (14.7)	74 (19.7)
I don't know if or when I should have a test	13 (12.0)	21 (20.0)	30 (18.4)	64 (17.0)
I have had a bad experience in the past having a test	29 (26.9)	17 (16.2)	15 (9.2)	61 (16.2)
I am not having sex	14 (13.0)	16 (15.2)	30 (18.4)	60 (16.0)
It is hard to find or get an appointment with the right nurse or doctor	9 (8.3)	11 (10.5)	8 (4.9)	28 (7.4)
It is hard to travel to an appointment	5 (4.6)	6 (5.7)	15 (9.2)	26 (6.9)
It is too expensive to have a test	7 (6.5)	4 (3.8)	9 (5.5)	20 (5.3)
I have never had sex	5 (4.6)	3 (2.9)	11 (6.7)	19 (5.1)
I have not received a reminder letter to have a test	5 (4.6)	3 (2.9)	11 (6.7)	19 (5.1)
It is hard to find a nurse or doctor of the right sex	10 (9.3)	3 (2.9)	6 (3.7)	19 (5.1)
My nurse or doctor has not suggested a test	2 (1.9)	1 (1.0)	10 (6.1)	13 (3.5)
It is hard to find a nurse or doctor who speaks my language	0 (0.0)	2 (3.1)	5 (1.9)	7 (1.9)
I have had a hysterectomy	3 (2.8)	1 (1.0)	3 (1.8)	7 (1.9)
It is hard to find a nurse or doctor of the right ethnicity	1 (0.9)	3 (2.9)	2 (1.2)	6 (1.6)
I don't think test results are accurate enough	0 (0.0)	4 (3.8)	1 (0.6)	5 (1.3)
Rather not say	3 (2.8)	4 (3.8)	2 (1.2)	9 (2.4)

*Note*: Multiple selections were possible.

^a^ Percentage is of all 376 participants, but multiple responses were allowed and included.

b The top three reasons were also the most common responses from the 176 women who provided a main reason for lack of attendance from a list of options.

^***^ *p* < .05 indicating a statistically significant effect of ethnicity on responses using a 3(ethnicity) × 2(selected reason or not) Pearson *χ* ^2^ test.

Forty‐one women provided a free‐text self‐reported reason for not having had a smear test (see the Supporting Information).

### 3.6 Future screening preferences

Women were asked to identify whether they would prefer to have a doctor or nurse do the test in the future or whether they would prefer to do it themselves and then to identify the two main reasons for choosing that answer. In total, 18 women (4.8%) indicated that they would prefer a doctor or nurse to take the test; 287 women (76.3%) indicated that they would prefer to take their own test at home; 63 women (16.8%) indicated that they would prefer to take their own test at a medical clinic; 3 women (0.8%) said they did not intend to do a test again; and 7 women (1.9%) did not know what they would prefer. 18 women left the question blank. The reasons for their preferences are presented in Supporting Information: Table S2, with the top reason for preferring a doctor or nurse to take the test being that the test is accurate and the top two reasons for preferring a self‐test being that it is less embarrassing and simple to do.

Women's preferences for receiving and returning the self‐test kit if they used it in the future are presented in Table 5. If the self‐test kit were to be mailed, 67.0% (*N* = 252) said that they would prefer the kit to be automatically sent the next time they were due for their smear test, whereas 23.4% (*N* = 88) said that they would prefer to receive a letter or call first; 2.1% (*N* = 8) said that they would prefer to order the kit online from a health professional. 28 women did not answer the question.

**Table 5:** Women's future preferences for receiving and returning the self‐test kit

Preference	Māori (%)	Pasifika (%)	Asian (%)	*N* a
Receive self‐test kit by mail***	83 (76.9)	64 (61.0)	124 (76.1)	271
Collect from clinic to do at clinic***	13 (12.0)	20 (19.0)	13 (8.0)	46
Collect from clinic to do at home	11 (10.2)	13 (12.4)	13 (8.0)	37
Collect from pharmacy to do at home	6 (5.6)	4 (3.8)	5 (3.1)	15
Collect from community laboratory to do at home	3 (2.8)	1 (1.0)	2 (1.2)	6
Return kit by courier***	49 (45.4)	28 (26.7)	74 (45.4)	151
Return kit to a clinic (such as family doctor)	30 (27.8)	39 (37.1)	49 (30.1)	118
Return kit to a community laboratory	22 (20.4)	20 (19.0)	28 (17.2)	70
Return kit to a pharmacy	11 (10.2)	6 (5.7)	11 (6.7)	28

^a^ NB Some women selected more than one response and some left the question blank; all responses were included.

*** *p*s < 0.05 indicating a statistically significant effect of ethnicity on responses using a 3(ethnicity) × 2(selected reason or not) Pearson *χ* ^2^ test.

## 4 DISCUSSION

The three main reasons Māori, Pasifika and Asian women in our study provided for not ever or not recently having a smear test were embarrassment, pain or discomfort and time. These findings are consistent with previous research,^9^, ^16^ which found that the primary barriers to conventional screening for Māori women were lack of time/other commitments, fear of discomfort or pain and that some Māori women consider this area to be tapu and therefore in some cases screening caused feelings of embarrassment. By contrast, most women in our study found the self‐test kit to be easy and convenient to use and reported that they did not find it painful, uncomfortable or embarrassing. This was also reflected in the preference for a self‐test over a future smear test on the grounds of ease, convenience, comfort and reduced embarrassment by most women who had previously had a smear test.

The only area of uncertainty around the self‐test kit was related to the perceived accuracy of the self‐test. A substantial minority (24%) were not confident or not sure that they had performed the test correctly. Furthermore, more than half the women (65%) who had previously had a smear test indicated that they were unsure whether the self‐test kit or smear test was more accurate. In a focused literature review exploring the acceptability, feasibility and uptake of HPV self‐testing among so‐called hard‐to‐reach women across the world between 1997 and 2015, a key barrier to self‐testing reported was a concern about sampling accuracy, including performing the procedure correctly.^17^ This barrier was reiterated in a more recent scoping review of research exploring HPV self‐testing in Indigenous communities.^18^ The provision of more information addressing these areas may allay women's concerns. Most women in our study (76%) indicated that they would prefer to take a self‐test at home or in a clinic (17%) rather than having a clinician do the test (5%) and the main reasons for this were reduced embarrassment, the simplicity of the test and not needing an appointment.

It is important to note that some of the findings differed by ethnicity. For example, although the three main reasons for not ever or not recently having had a smear test were the same for each group, the percentage of women from each group who reported these differed. For example, more Māori women than Pasifika women and more Pasifika women than Asian women reported embarrassment or pain or discomfort as a reason, while more Asian women than Māori or Pasifika women reported time as a reason. These and the other differences should be used to inform the design of information and educational materials to support HPV primary screening and self‐testing that target at‐risk groups.

The findings highlight areas where more information may be useful to increase uptake. For example, most women in our survey did not know whether the self‐test or smear test was more accurate, 20% did not think they needed a test and, relatedly, 16% reported not having the test because they were not having sex. Our findings also provide some clear insights into women's preferences, which should be used to inform the implementation of HPV primary screening and self‐testing. Most women (72%) would prefer to receive a self‐test kit mailed to their home, although some women would prefer to collect it from a clinic to do at the clinic (12%) or at home (10%). Importantly, if the test were to be mailed to their home, although most women (67%) would be happy for this to happen automatically, a sizable minority (23%) would prefer to receive a letter or call first. In relation to returning the kit, findings were more mixed. Some women would prefer to return the test by courier, some would prefer to return it to a clinic and others to a pharmacy or community laboratory. A range of options would provide the best rate of return, which is consistent with research that showed that a community laboratory drop‐off alternative to postal return increased participation in New Zealand's Bowel Screening Pilot.^19^ As HPV self‐testing is introduced, it will be important that these preferences are taken into account to maximize uptake of self‐testing. A new purpose‐built NCSP population register should allow recording of preferences for invitation and recall.

Maximizing access to self‐testing can also be informed by our phone calls with nonresponders, which revealed that, although most had received the test kit, there were a variety of reasons for not doing the test, including not wanting to do it, being too busy or forgetting (see the Supplementary Materials). Follow‐up phone calls were very time‐consuming, so further research could investigate ways to maximize uptake of unsolicited self‐testing kits, possibly with the use of advance notification that the kit was being sent and follow‐up reminder letters, texts or calls.

The aforementioned scoping review of research exploring HPV self‐testing in Indigenous communities covered the years 1993 to 2018 and just 19 studies, including grey literature, fulfilled the criteria^18^; however, none of these were from Aotearoa New Zealand. The study reported here builds on previous research,^10^, ^16^ as well as our own feasibility study^12^ to provide the first research that explores the preferences around HPV self‐testing of Māori, Pasifika and Asian women who have actually used a self‐testing kit. This study is timely for three reasons. First, the Aotearoa New Zealand Government has recently announced that they will invest “up to $53 million to … implement a new human papillomavirus (HPV) test” in 2023.^20^ The screening programme will introduce the option of either a self‐test or clinician‐taken sample to all women, which means that it is crucial that culturally relevant research and Māori, Pasifika and Asian expertise and knowledge are used to inform the design and roll‐out of the new programme. Second, HPV self‐testing has recently been announced as an option for participants in the Australian National Screening Programme from July 2022.^21^ Third, a recent editorial^22^ further suggests that the negative impact of the COVID‐19 pandemic on cervical screening uptake may prove to be a tipping point for the wider introduction of self‐testing. It is likely that Indigenous groups in other countries can benefit from these Aotearoa New Zealand findings.

To achieve elimination of cervical cancer as characterized by the WHO (4 cases per 100,000 women^23^), incidence must be reduced by 63% for Māori women.^24^ This needs to be achieved by a concerted effort across primary care through HPV vaccination, cervical screening, diagnosis and treatment/follow‐up pathways. Our findings indicate that HPV self‐testing would offer an acceptable and culturally appropriate addition to the screening programme in Aotearoa New Zealand for Māori, Pasifika and Asian women – particularly if the preferences identified in our research are implemented – and thus has considerable potential to reduce inequities in access to screening and to save lives. Now that the Government has committed to rolling out HPV primary testing inclusive of self‐testing, equity‐focused approaches are required to ensure that benefits address the current and longstanding inequities in access to cervical screening and cervical cancer outcomes in Aotearoa New Zealand.

## AUTHOR CONTRIBUTIONS

**Karen Bartholomew**: Conceptualization, funding acquisition, investigation, methodology, project administration, supervision, writing – review and editing. **Naomi Brewer**: Conceptualization, data curation, funding acquisition, investigation, methodology, project administration, supervision, writing – review and editing. **Collette Bromhead**: Conceptualization, funding acquisition, investigation, methodology, project administration, supervision, writing – review and editing. **Jeroen Douwes**: Conceptualization, funding acquisition, investigation, methodology, project administration, resources, supervision, writing – review and editing. **Anna Maxwell**: Conceptualization, investigation, methodology, project administration, supervision, writing – review and editing. **John D. Potter**: Conceptualization, funding acquisition, investigation, methodology, project administration, resources, supervision, writing – review and editing. **Susan M. Sherman**: Formal analysis, investigation, roles/writing – original draft, writing – review and editing. **Chris Cunningham**: Funding acquisition, investigation, methodology, writing – review and editing. **Sue Crengle**: Investigation, writing – review and editing. **Sunia Foliaki**: Funding acquisition, Investigation, writing – review and editing. **Jane Grant**: Conceptualization, investigation, writing – review & editing. **Georgina McPherson**: Funding acquisition, investigation, writing – review and editing. **Nina Scott**: Methodology, writing – review and editing. **Helen Wihongi**: Funding acquisition, investigation, methodology, writing – review and editing.

## CONFLICTS OF INTEREST

Naomi Brewer reports grants from Janssen‐Cilag Pty Limited, outside the submitted work. Collette Bromhead has previously received educational funding to attend conferences from Roche Diagnostics New Zealand. Sue Crengle reports personal fees from Board member WellSouth Primary Health Network, personal fees from General Practitioner, Invercargill Medical Centre, personal fees from Board member, Royal NZ College of General Practitioners, outside the submitted work. H Wihongi reviews grants and other research applications from Waitematā District Health Board, outside the submitted work. She is also a member and convener of the National Kaitiaki Group. The remaining authors declare no conflict of interest.

## ETHICS STATEMENT

The study was approved by the Aotearoa New Zealand Northern B Health and Disability Ethcis Committee (HDEC) (reference: 17/NTB/120). The Aotearoa New Zealand Ministry of Health (including the National Kaitiaki Group—a group of Māori women established to monitor the safe use of Māori data within the NCSP) and the participating DHBs, Primary Health Organizations and clinics approved the use of data to identify and contact eligible women.

## TRANSPARENCY DECLARATION

The authors affirm that the manuscript is an honest, accurate, and transparent account of the study being reported; that no important aspects of the study have been omitted; and that any discrepancies from the study as originally planned have been explained.

## Supporting information

Supporting information.

## Data Availability

As a result of ethics requirements and issues around data sovereignty associated with Indigenous people, at present, we are unable to share data.

## Footnotes

* Pasifika (sometimes spelt Pasefika) is used to refer to the people, cultures and language of Pacific groups including Sāmoa, Tonga, the Cook Islands, Niue, Tokelau, Tuvalu and other smaller Pacific nations—who are now living in Aotearoa New Zealand. We acknowledge that there is ‘no generic “Pacific community”’ and that the term Pasifika is ‘a category defined by New Zealand policy and discourse’.^5^

^†^
Whereas whakamā has been used in the past (including in the study we refer to here)^9^ and is still relevant in some cases, the term tapu is the preferred term. Tapu acknowledges the mana (power), wairua (connection to the spirit world) and mauri (essence) of Māori women. There is critique of the term whakamā (a feeling of being at a disadvantage, often a cultural disadvantage) in the interpretation of women's reporting about the invasiveness of vaginal examinations/cytology test, with the use of these terms seen to be victim blaming/deficit framing. We deliberately refer here to the invasive nature of the test to reflect that it is the test that is problematic.

^‡^
While both self‐testing and self‐sampling are used in the literature, self‐testing was found to be the term preferred by women. Accordingly, that is the terminology we have used throughout the manuscript.
