# Supplementary material for: Human papillomavirus self‐testing among unscreened and under‐screened Māori, Pasifika and Asian women in Aotearoa New Zealand: A preference survey among responders and interviews with clinical‐trial nonresponders
Source: Health Expect. 2022 Sep 26;25(6):2914–23. doi: 10.1111/hex.13599 (PMC9700139; doi:10.1111/hex.13599)
Supplement: Supplementary file 1 — Supporting information. [file HEX-25--s001.docx]

**Supplementary Materials.**

Additional data referenced in the text is presented here with relevant section headings from the main text.

**Using the self-test kit**

Forty-two women provided responses other than N/A or ‘I didn’t watch them’ to the open-ended question asking for comments about the video clips. The most frequent response was that they were clear and easy to understand (N=18). Eight women found the written or nurse instructions sufficient. Four women found them reassuring, four were too busy to watch them, two women were still uncertain about the test, two women commented on the different languages (one positive, one suggesting they could be more than just subtitles) and the rest of the responses mainly concerned technical issues (e.g., “Because video clips not available”).

**Table S1. Women’s opinion of the self-test kit compared with a previous smear test**

Women who had previously had a smear test were asked to compare this with the self-test kit and their responses are presented below.

|  | **Self-test** | **Smear test** | **No difference** | **Unsure/don’t know** | **Total N** |
| --- | --- | --- | --- | --- | --- |
| **Easier**  Māori  Pasifika  Asian  Total | 81 (92.0)  57 (80.3)  70 (79.5)  208 (84.2) | 2 (2.3)  6 (8.5)  6 (6.8)  14 (5.7) | 4 (4.5)  4 (5.6)  7 (8.0)  15 (6.1) | 1 (1.1)  4 (5.6)  5 (5.7)  10 (4.0) | 88  71  88  247 |
| **More convenient**  Māori  Pasifika  Asian  Total | 83 (96.5)  56 (88.9)  75 (89.3)  214 (91.8) | 1 (1.2)  4 (6.3)  3 (3.6)  8 (3.4) | 1 (1.2)  1 (1.6)  3 (3.6)  5 (2.1) | 1 (1.2)  2 (3.2)  3 (3.6)  6 (2.6) | 86  63  84  233 |
| **Less embarrassing*****  Māori  Pasifika  Asian  Total | 81 (90.0)  57 (90.5)  69 (84.1)  207 (88.1) | 2 (2.2)  4 (6.3)  0 (0.0)  6 (2.6) | 6 (6.7)  2 (3.2)  9 (11.0)  17 (7.2) | 1 (1.1)  0 (0.0)  4 (4.9)  5 (2.1) | 90  63  82  235 |
| **Less uncomfortable**  Māori  Pasifika  Asian  Total | 79 (90.8)  55 (88.7)  67 (77.0)  201 (85.2) | 3 (3.4)  5 (8.1)  6 (6.9)  14 (5.9) | 4 (4.6)  2 (3.2)  10 (11.5)  16 (6.8) | 1 (1.1)  0 (0.0)  4 (4.6)  5 (2.1) | 87  62  87  236 |
| **More accurate*****  Māori  Pasifika  Asian  Total | 15 (18.5)  22 (38.6)  11 (13.6)  48 (21.9) | 4 (4.9)  8 (14.0)  5 (6.2)  17 (7.8) | 5 (6.2)  1 (1.8)  5 (6.2)  11 (5.0) | 57 (70.4)  26 (45.6)  60 (74.1)  143 (65.3) | 81  57  81  219 |

*** p <.05 indicating a statistically significant effect of ethnicity on responses using a 3(ethnicity) x 4(response type) Pearson Chi Squared test.

**Barriers to smear test**

Forty-one women provided a free text self-reported reason for not having had a smear test. These included dislike of test or fear of what might be found or needed as next steps (N=6), personal or relative’s previous bad experience of a smear test (N=6), shyness, embarrassment or modesty (N=6), too busy working, not bothered or living elsewhere (N=5), current or recent pregnancy (N=5), recently had the test or felt that the current schedule is too frequent (N=4), believing that they had not had exposure to HPV or didn’t need the test because they were a lesbian (N=3) and 6 miscellaneous reasons (e.g., looking for new doctor).

**Table S2. Reasons for future screening preferences**

Reasons for preferring future tests to be administered by a doctor/nurse or self-administered (women were invited to select two reasons).

| **Reasons for preferring a doctor or nurse to take the test** | **Māori (%)** | **Pasifika (%)** | **Asian (%)** | **N** |
| --- | --- | --- | --- | --- |
| The test is accurate | 5 (4.6) | 7 (6.7) | 18 (11.0) | 30 |
| I can ask the nurse or doctor about something else*** | 1 (0.9) | 3 (2.9) | 13 (8.0) | 17 |
| The test is simple to do | 4 (3.7) | 5 (4.8) | 7 (4.3) | 16 |
| The test may find other problems | 3 (2.8) | 6 (5.7) | 7 (4.3) | 16 |
| The test is convenient | 2 (1.9) | 2 (1.9) | 6 (3.7) | 10 |
| The test is less embarrassing | 4 (3.7) | 1 (1.0) | 1 (0.6) | 6 |
| **Reasons for preferring to take the test yourself** |  |  |  |  |
| The test is less embarrassing | 61 (56.5) | 56 (53.3) | 75 (46.6) | 193 |
| The test is simple to do | 60 (55.6) | 52 (49.5) | 77 (47.2) | 189 |
| I do not need an appointment with a nurse or doctor to do the test | 54 (50.0) | 43 (41.0) | 87 (53.4) | 184 |
| The test does not require the use of instruments (e.g., speculum)*** | 48 (44.4) | 29 (27.6) | 47 (28.8) | 124 |
| The test is free | 25 (23.1) | 28 (26.7) | 48 (29.4) | 101 |
| The test is accurate | 4 (3.7) | 6 (5.7) | 4 (2.5) | 14 |

*** p <.05 indicating a statistically significant effect of ethnicity on responses using a 3(ethnicity) x 2(selected reason or not) Pearson Chi Squared test

**Table S3. Codes generated by content analysis of the interviews with non-responders.**

| **Code name** | **Number of comments per code** | **Indicative quotes or comments by interviewer** |
| --- | --- | --- |
| Received pre-invite or kit | 19 | Received the invitation, forgot to complete her test. Found the information very helpful and clear. The mobile number we had was invalid, so she didn’t know about the opportunistic invitation [for the sub-study], however, wanted a kit to be sent out if possible. [Asian clinic]  Received all information and texts. She didn’t do the test as she doesn’t want to do it. When I asked if there was anything that would have encouraged her to she said no she just didn’t want to do it. [Pasifika home] |
| Wanted a kit to be sent | 7 | Never received the kit. ‘Sounds good’ if she doesn’t have to ‘have someone go down there’. Would like to have another look if not too late. Address updated and new kit sent out. [Pasifika home]  Received the invitation however never had time to go in and do the test. She said she thought it was a good idea if she could do it at home or the clinic but she ran out of time. I offered to send her a home kit and she was happy with that option. Contact details are all correct. [Pasifika clinic] |
| Information was good/liked the idea | 6 | The information was very helpful but with work and family had not got around to it yet. [Asian clinic]   Found the information was good just didn’t want to take part and forgot to ring. [Māori home] |
| Does not want to do the test | 6 | Received all information and texts. She did not do the test as she does not want to do it. When I asked if there was anything that would have encouraged her to, she said no she just did not want to do it. [Pasifika home]  She said she just wanted to ignore doing it. [Asian clinic] |
| Threw kit away/lost it | 5 | “I have limited movement in my arms and neck … I was a bit angry when I got the invite as how the hell am I going to do that? So I threw it away and didn’t bother with it again” [Māori home]  When she got home she thought she had missed it so just threw it away. “I wasn’t in NZ when the information was sent - I thought I had missed out. It would have been better if you had emailed me the information” [Pasifika home] |
| No invite received | 4 | Never received the information. [Māori home]  Never received the kit. ‘Sounds good’ if she doesn’t have to ‘have someone go down there’. Would like to have another look if not too late. Address updated and new kit sent out. [Pasifika home] |
| Too busy to do the test | 4 | Received the invitation but has been busy with work and home. The information was very helpful but with work and family hadn’t gotten around to it yet. [Asian clinic]  Received the invitation however never had time to go in and do the test. She said she thought it was a good idea if she could do it at home or the clinic but she ran out of time. [Pasifika clinic] |
| Forgot to do the test | 3 | Went into clinic and the nurse explained everything to her, she said it looked easy and simple. She took a kit home to do the test and life got busy and she forgot. However, because I called she will do the test as soon as possible and send it back in the courier bag. [Pasifika clinic]  Received the invitation and thought it was something she should do then put the information somewhere and forgot. She asked to get a home kit and if she is still unsure she will take it to the GP clinic and do it there. [Asian clinic] |
| Didn’t understand what it was/poor English | 3 | Did not understand English so spoke to young son who translated. [Asian clinic]  Didn’t really understand what the test was for (not very good English). [Asian home] |
| Doesn’t want GP or nurse to do the test | 3 | “I didn’t keep the letter, I get embarrassed and don’t want the nurse or doctor going there. If I can do it myself then I will try.” [Pasifika clinic]  Also didn’t want the Dr or nurse to do that either. [Asian home] |
| Never had sex | 3 | Has never had sex so didn’t do the test and didn’t think she needed to call. [Asian clinic]  Participant never had sex before and had told her Drs that when they asked ‘why didn’t they tell us that so not to waste time, why ask me if people don’t put it on my file’. [Pasifika home] |
| Will do test herself | 3 | Yes, she knows about the study and yes she will do it when she gets around to it. [Māori clinic]  After I explained the test was happy to do it as it was ‘a lot easier and less drama than going to the Drs’. [Māori home] |
| Will take test to GP | 2 | Still has the kit and information so will take with her to the doctor. [Asian home]  Wants to do a test at the Drs not at home - not sure if safe to do at home as never done anything like that before. [Asian home] |
| Will go for normal smear test | 2 | Participant lost the kit when moving house (before Christmas). Wasn’t really interested as too busy. Will sort out doing her smear when she has more time. [Māori home]  Not really interested (not her thing) will just have a smear done next time. [Māori home] |
| Sacred body/embarrassment | 2 | “was raised that this is a sacred part of my body and to be treated with respect. I have had smears in the past and was uncomfortable with the invasiveness of them. ... if I feel the need, I will seek what help I want.” [Māori clinic]  “I didn’t keep the letter, I get embarrassed and don’t want the nurse or doctor going there. If I can do it myself then I will try.” [Pasifika clinic] |
| Invasive procedure | 2 | Didn’t like the idea of ‘sticking anything up there’. [Asian home]  “[-] this is an invasive process not just self-sampling but smears as well [-]” [Māori clinic] |
| Will seek help if needed | 2 | “[-]I have had smears in the past and was uncomfortable with the invasiveness of them. I understand that this is just my view. I am not interested in any of these initiatives and if I feel the need I will seek what help I want. [-]” [Maori clinic]  She asked to get a home kit and if she is still unsure she will take it to the GP clinic and do it there. [Asian clinic] |
| Other responses (each N=1) | 8 | Received the pack for smear test but is currently 7 months pregnant. [Asian home]  Patient wasn’t in NZ when her study information arrived. When she got home thought she had missed it so just threw it away. ‘I wasn’t in NZ when the information was sent I thought I had missed out. It would have been better if you had emailed me the information. [Pasifika home] |
